# Retinal Proteome Analysis Reveals a Region-Specific Change in the Rabbit Myopia Model

**DOI:** 10.3390/ijms24021286

**Published:** 2023-01-09

**Authors:** Chae-Eun Moon, Yong Woo Ji, Jun-ki Lee, Kyusun Han, Hyunjin Kim, Seok Ho Byeon, Suenghan Han, Jinu Han, Yuri Seo

**Affiliations:** 1 Institute of Vision Research, Department of Ophthalmology, Yonsei University College of Medicine, Seoul 03722, Republic of Korea; 2 Department of Ophthalmology, Graduate School of Medical Science, Brain Korea 21 Project, Yonsei University College of Medicine, Seoul 03772, Republic of Korea; 3 Institute of Vision Research, Department of Ophthalmology, Yongin Severance Hospital, Yonsei University College of Medicine, Yongin-si 16995, Republic of Korea; 4 Institute of Vision Research, Department of Ophthalmology, Severance Hospital, Yonsei University College of Medicine, Seoul 03722, Republic of Korea; 5 Institute of Vision Research, Department of Ophthalmology, Gangnam Severance Hospital, Yonsei University College of Medicine, Seoul 03722, Republic of Korea

**Keywords:** myopia, proteomics, retina, oxidative stress, coagulation, regional

## Abstract

Uncovering region-specific changes in the myopic retina can provide clues to the pathogenesis of myopia progression. After imposing form deprivation myopia in the right eye of 6-week-old rabbits, we investigated the proteome profile of each retinal region (central, mid-periphery, and far-periphery retina), using accurate high-resolution mass spectrometry. Protein expression was analyzed using gene ontology and network analysis compared with that of the control, the left eyes. Among 2065 proteins detected from whole retinal samples, 249 differentially expressed proteins (DEPs) were identified: 164 DEPs in the far-periphery, 39 in the mid-periphery, and 83 in the central retina. In network analysis, the far-periphery retina showed the most significant connectivity between DEPs. The regulation of coagulation was the most significant biological process in upregulated DEPs in the far-periphery retina. Proteasome was the most significant *Kyoto Encyclopedia of Genes and Genomes* pathway in downregulated DEPs in the central retina. Antithrombin-III, fibrinogen gamma chain, and fibrinogen beta chain were identified as hub proteins for myopia progression, which were upregulated in the far-periphery retina. Proteomic analysis in this study suggested that oxidative stress can be the primary pathogenesis of myopia progression and that the far-periphery retina plays a role as the key responder.

## 1. Introduction

Oxidative stress and chronic inflammation have gained importance in the pathogenesis of myopia. The prevalence of myopia is increased in inflammatory diseases, such as diabetes, systemic lupus erythematous, and allergic disease [1,2]. The expression levels of pro-inflammatory cytokines, such as nuclear factor kappa B, interleukin 6, and tumor necrosis factor alpha, increased in the myopic sclera and decreased after treatment with atropine [1]. Hypoxia-signaling pathways were activated in the murine myopic sclera, and hypoxia exposure promoted myofibroblast transdifferentiation with the downregulation of type 1 collagen in human scleral fibroblasts [3]. In pathologic myopia patients, the levels of antioxidants (glutathione peroxidase 3 and prostaglandin H2-D-isomerase) were significantly decreased in vitreous humor compared with those in the controls [4].

The retina is one of the primary oxygen-consuming tissues regulated by vascular supply and its alterations [5]. Additionally, the retina is metabolically active, making it particularly vulnerable to oxidative stress [6]. The role of the retina in myopia has not yet been fully determined. However, it becomes significantly thinner after the induction of myopia. Eric B. et al. [7] demonstrated that the upregulation of apoA-I in the retina was a STOP signal for myopia progression.

In myopia progression, axial elongation has been related to the thinning of the retina and the reduction in retinal pigment epithelium (RPE) of the peripheral area in emmetropization. In contrast, the thinning of RPE density or retinal thickness has not been observed in the macular area [8]. The underlying cause of this region-specific retinal change has not been elucidated. In the human eye, previous studies have investigated region-specific characteristics of the retina. Gabriel V. et al. reported that the retinal regions’ susceptibility to metabolic and oxidative stress differs [9]. However, the study was performed in the aged retina; hence, the effect of aging cannot be excluded. In addition, the refraction status of samples was not well described.

In this study, we analyzed the region-specific retinal proteome profile after myopia induction in a rabbit model using high-resolution accurate mass (HRAM) mass spectrometry. Additionally, we investigated myopia-specific hub proteins in each retinal region, comparing proteomes between control and myopia-induced eyes. It was expected that a more comprehensive understanding of myopia progression mechanisms could be achieved by clarifying proteomic changes in each retinal region.

## 2. Results

### 2.1. Effect of Form Deprivation on Refraction and Axial Length

**Figure 1:**
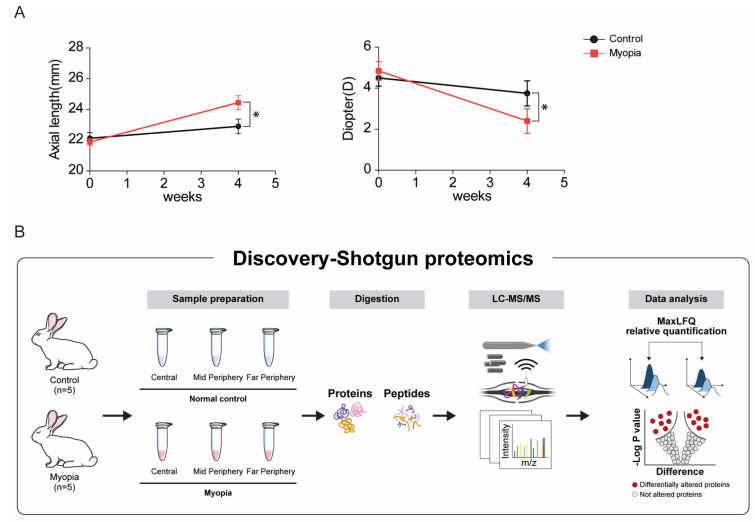
Myopia induction and proteomic analysis of myopic retina in each retinal region: (**A**) to verify the induction of myopia, refraction and axial length were measured (* *p* < 0.01); (**B**) using LC-MS/MS with MaxQuant processing, 2065 proteins in total were identified from the whole retinal sample.

We compared the axial length and refraction between the myopic and control eyes. After 6 weeks of form deprivation, the axial length of the myopia-induced eye significantly increased more than that of the control eye (baseline: control = 22.13 ± 0.83 mm, myopia-induced = 21.88 ± 0.52 mm; after myopia induction: control = 22.9 ± 1.08 mm, myopia-induced = 24.45 ± 1.01 mm; repeated measures analysis of variance (ANOVA) test, *p* < 0.01, control vs. myopia-induced eyes, Figure 1A). Form deprivation induced more myopic refractive change in the myopia-induced eyes than that in the control eyes (baseline: control = 4.5 ± 0.39 D, myopia-induced = 4.85 ± 0.45 D; after myopia induction: control = 3.75 ± 0.61 D, myopia-induced = 2.40 ±0.6 D; repeated measures ANOVA test, *p* < 0.01, control vs. myopia-induced eyes, Figure 1A).

### 2.2. Total Proteomic Profiling of Retinal Tissues from the Myopia-Induced Eye and Control Eye

We segmented each retina in the myopia-induced and control eyes into the central, mid-periphery, and far-periphery retina. Proteins from six groups were analyzed using LC-MS/MS following trypsin digestion followed by processing with MaxQuant. A total of 2065 proteins were identified from the whole retinal sample (Figure 1B).

**Figure 2:**
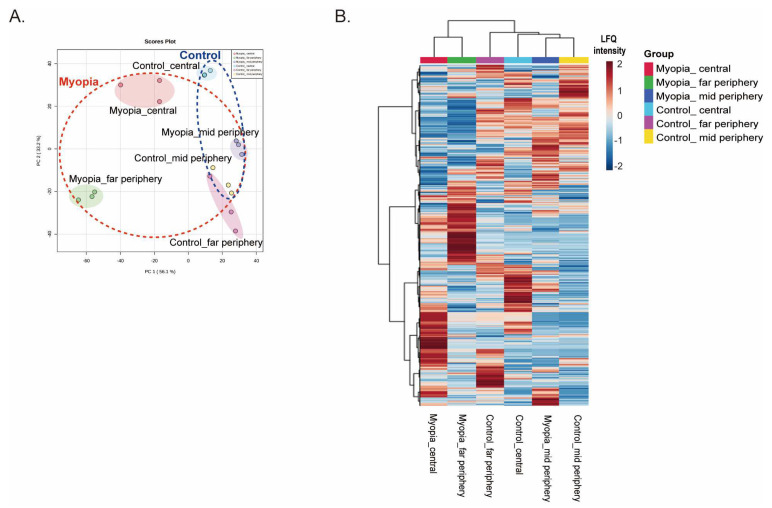
Hierarchical clustering of expressed proteins in each retinal region: (**A**) principal component analysis (PCA) demonstrated that far-periphery myopia retinas and far-periphery control retinas were distinguished; on the other hand, the mid-periphery myopia retinas and control retinas were not differentiated. Central-myopia retinas and control retinas were well distinguished on PCA; (**B**) unsupervised hierarchical clustering of the whole retinal proteome generated a heat map similar to PCA.

We compared each of the six groups using principal component analysis (PCA) (Figure 2A). PCA distinguished far-periphery myopia retina and far-periphery control retina; however, the mid-periphery retina was similar when comparing myopia and control retinas. PCA also distinguished central myopia retina and central control retina but not as much as the far-periphery retina. Unsupervised hierarchical clustering of whole proteomes was generated as a heat map by the regional groups (Figure 2B). The most abundant and accumulated proteins in the far-periphery myopia retina were at reduced levels in the far-periphery control retina. The far-periphery myopia retina was clustered with the central myopia retina. In comparison, the mid-periphery control retina and the mid-periphery myopia retina were clustered in the same group.

### 2.3. Identification of DEPs in Each Retinal Region after Myopia Induction

**Figure 3:**
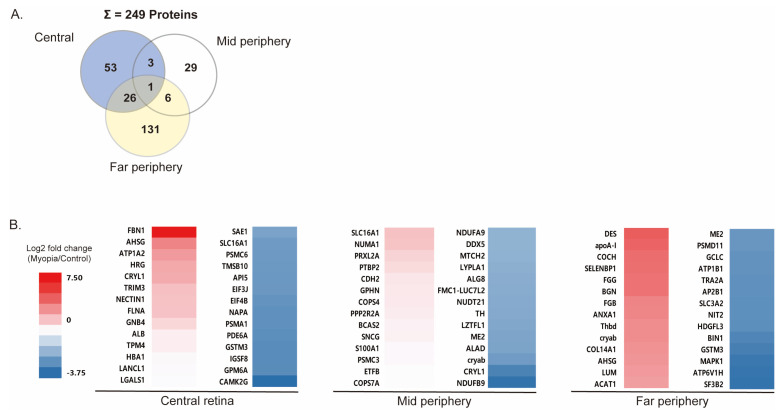
Differentially expressed proteins (DEPs) in each retinal region: (**A**) in total, 249 DEPs were identified from whole retinal regions; in total, 164 DEPs were identified in the far-periphery retina occupying the largest proportion of DEPs, followed by the central retina (83 DEPs) and the mid-periphery retina (39 DEPs); (**B**) heatmap of DEPs in each retinal region; the box color indicates log2 fold changes of DEPs from red (increasing) to blue (reducing), when comparing a myopic eye to a control eye.

After myopia induction, 249 DEPs were identified in the whole retina (Figure 3A). The expression pattern of DEPs was well differentiated between each retinal region (Figure S1). In the central retina, 83 DEPs were identified in total: 16 upregulated proteins (UP-DEPs) and 67 downregulated proteins (DOWN-DEPs). In the mid-peripheral retina, 39 DEPs—21 UP-DEPs and 18 DOWN-DEPs—were identified. In the far-peripheral retina, 164 DEPs—83 UP-DEPs and 81 DOWN-DEPs—were identified (Table S1). The most abundant DEPs were identified in the far-periphery retina (Figure 3B). The top 14 UP-DEPs and DOWN-DEPs are shown in Figure 3B. In the central retina, the expression of fibrillin 1 (FBN1) increased by 164.03 fold changes (FCs, *p* < 0.01), followed by that of alpha-2-HS-glycoprotein (AHSG, FC = 16.34, *p* = 0.03), sodium/potassium-transporting ATPase subunit alpha (ATP1A2, FC = 11, *p* < 0.01), histidine-rich glycoprotein (HRG, FC = 8.93, *p* < 0.01), and lambda-crystallin (CRYL1, FC = 8.4, *p* < 0.01). In the far-periphery retina, DEPs showed, on an average, the most significant fold changes compared with those at other regions (Figure 3B). The expression levels of Desmin (DES, FC = 34.5, *p* < 0.01), apolipoprotein A-1 (apoA-1, FC = 30.61, *p* < 0.01), cochlin (COCH, FC = 24.82, *p* < 0.01), and selenium binding protein 1 (SELENBP1, FC = 23.97, *p* < 0.01) increased in the far-periphery. 

### 2.4. Proteome Interaction Network Model Describing Myopia Induction

**Figure 4:**
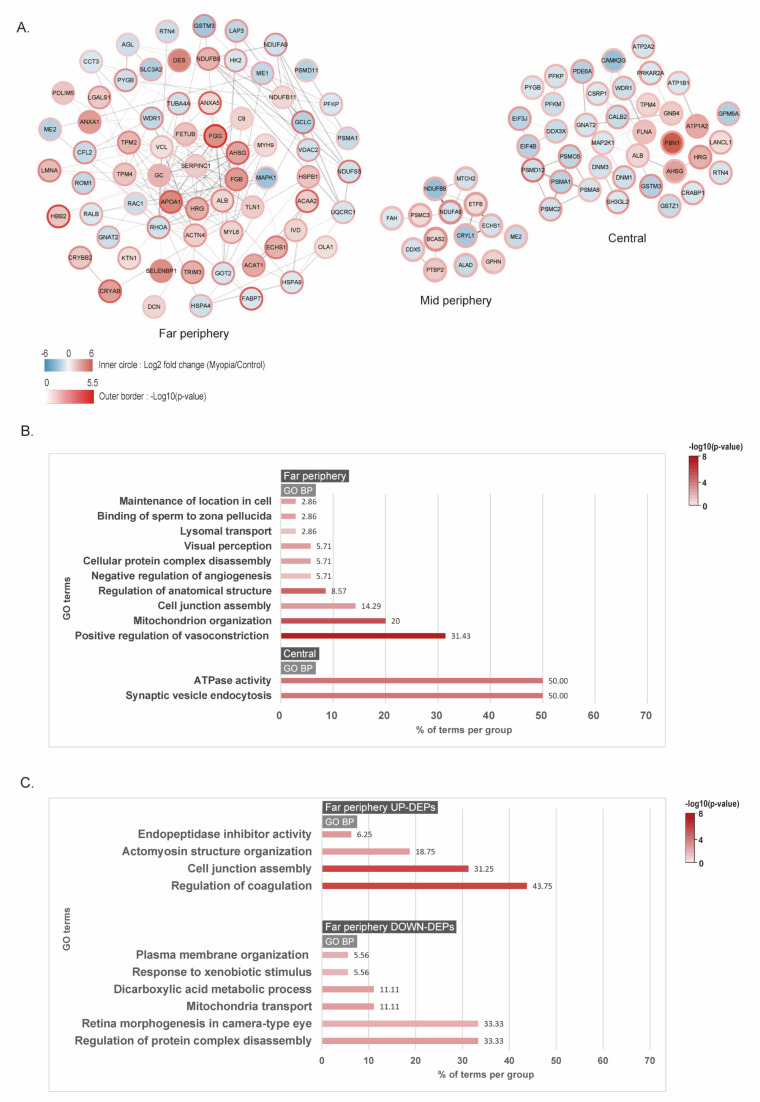
Network modeling of differentially expressed proteins in each region of myopia versus control retina: (**A**) protein–protein interaction network of DEPs in each retinal region (node color: −log2 (fold changes); border color: –log10 (*p*-value); thickness of edge: connectivity between nodes); (**B**) gene ontology (GO) analysis of whole DEPs with biological process terms in the far-periphery and central retina; (**C**) GO analysis of upregulated and downregulated DEPs in the far-periphery retina with biological process terms.

We constructed protein–protein interaction (PPI) network models using DEPs from each regional retina to identify the essential proteins involved in critical biological processes for myopia induction (Figure 4A). Using the STRING database, nodes with a score ≥ 0.4 were selected to build the PPI network. Node color indicated fold changes in DEP expression after myopia induction by comparing the same retinal regions of the control. FC was converted to log2 values ranging from −6 (blue) to 6 (red). The node border color revealed a *p*-value, which was transformed to –log10 values ranging from 0 (white) to 5.5 (dark red). Closer connectivity between each node was presented by more edges with a thicker width. The connectivity was the most pronounced in the far-periphery retina (Figure 4A).

### 2.5. Functional Analysis of the DEPs in the Retina after Myopia Induction

To analyze the changes in DEPs after myopia induction, gene ontology (GO) was searched to investigate the biological processes and cellular components. *Kyoto Encyclopedia of Genes and Genomes* (KEGG) pathway annotation was also performed for each retinal region. Our results showed that the far-periphery retina mainly plays a role in changing DEPs after the induction of myopia. For whole DEPs, the most important biological processes were the positive coagulation of vasoconstriction, mitochondrion organization, and cell junction assembly on the far-periphery retina (Figure 4B). ATPase activity and synaptic vesicle endocytosis were involved in the change in the central retina (Figure 4B). The mid-periphery retina did not show any definite GO term because of a low p-value. For UP-DEPs on the far-periphery retina, the most significant biological process was the regulation of coagulation and cell junction assembly (Figure 4C). For DOWN-DEPs on the far-periphery retina, the most significant biological processes were the regulation of protein complex disassembly and retina morphogenesis in the camera-type eye (Figure 4C). On the central retina, no definite GO term was identified with UP-DEPs. DOWN-DEPs of the central retina involved biological processes such as the synaptic vesicle cycle and ATPase activity coupled to the transmembrane movement of ions and phosphorylation (Figure S2). Proteasome was the most significant KEGG pathway in DOWN-DEPs of the central retina.

### 2.6. Identification of Biomarkers for Myopia Progression in Each Retinal Region

**Figure 5:**
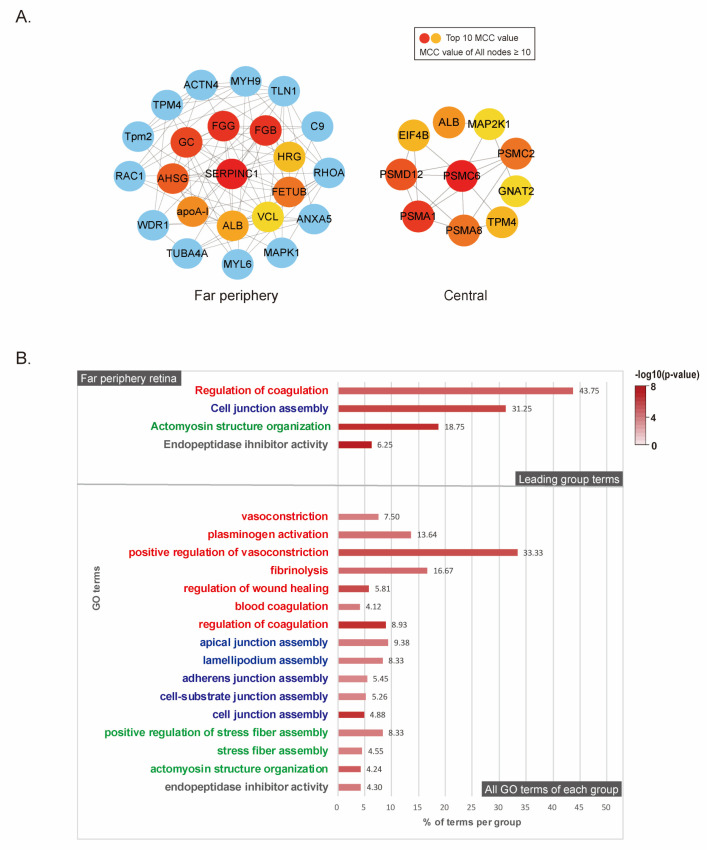
Hub protein identification based on MCC values and its GO analysis: (**A**) identification of hub proteins using the CytoHubba plugin; the maximal clique centrality (MCC) score was calculated, and nodes with the top 10 MCC values were identified as hub proteins colored yellow to red. All nodes with MCC value ≥ 10 were included in the GO analysis. MCC values of blue nodes were equal to or more than 10, but not in the top 10 values; (**B**) GO analysis with hub proteins whose MCC value was ≥10.

We screened the hub proteins using the maximal clique centrality (MCC) algorithm using the CytoHubba plugin. The proteins with the top 10 MCC values were considered hub proteins (Figure 5A). In the far-periphery retina, antithrombin-III (SERPINC1), fibrinogen gamma chain (FGG), fibrinogen beta chain (FGB), Gc-globulin (GC), alpha-2-HS-glycoprotein (AHSG), fetuin B (FETUB), apolipoprotein A-I (APOA1), albumin (ALB), histidine-rich glycoprotein (HRG), and metavinculin (VCL) were identified as hub proteins with the top 10 MCC values. In the central retina, proteasome 26S subunit, ATPase 6 (PSMC6), proteasome subunit alpha type (PSMA1), proteasome 26S subunit, non-ATPase 12 (PSMD12), proteasome 20S subunit alpha 8 (PSMA 8), 26S proteasome AAA-ATPase subunit RPT1 (PSMC2), albumin (ALB), eukaryotic translation initiation factor 4B (EIF4B), tropomyosin 4 (TPM4), G protein subunit alpha transducin 2 (GNAT2), and dual-specificity mitogen-activated protein kinase 1 (MAP2K1) were selected as hub proteins with the top 10 MCC values. Proteins presenting MCC values greater than 10 were included in the GO analysis of the biological process (Figure 5A, top 10 MCC nodes: red, orange, yellow; nodes with MCC values greater than 10 but not in the top 10: blue). Mid-periphery retinas did not reveal any protein with MCC values more than 10. On far-periphery retinas, the most significant biological processes were the regulation of coagulation, cell junction assembly, actomyosin structure organization, and endopeptidase inhibitor activity. These GO terms were group-leading terms which were the most significant term from the group. The–log10 (*p*-value) significance was visualized with color from 0 (white) to 8 (dark red). Detailed GO terms in each group were shown in the same color (Figure 5B). The result was consistent with the GO analysis of UP-DEPs in the far-periphery retina (Figure 4C). No GO terms were found on the central retina. Therefore, the top three hub proteins were selected in far-periphery retina. SERPINC1, FGG, and FGB were enriched as the main seeds of the network with the highest MCC score (SERPINC1: 2190, FGG: 2184, FGB: 2184). 

## 3. Discussion

Here, we have reported three main findings that are distinct from previous studies: (1) proteomic changes in retinal protein were the most significant in the far-periphery retina after myopia induction, followed by the central retina; (2) coagulation regulation, complement, and coagulation cascades were the most significant and upregulated biological process for myopia induction in the far-periphery retina; and (3) antithrombin-III (SERPINC1), fibrinogen gamma chain (FGG), and fibrinogen beta chain (FGB) are potential novel biomarkers for myopia induction in the retina.

The regulation of coagulation was enriched as the most significant biological process in UP-DEPs of the far-periphery retina. The result was consistent with GO analysis for hub proteins in the far-periphery retina. This consistency can confirm the importance of coagulation regulation as a key biological process of myopia induction. Hub proteins with the top 10 MCC values in this study included SERPINC1, FGB, APOA1, AHSG, and HRG, which had been identified in previous studies using human aqueous humor of pathologic myopia; however, Xu et al. had reported that APOA1 was the most significant hub protein [10]. In addition, liquid vitreous of LRP2 myopic mice showed increased complement and coagulation pathways, including SERPINC1, APOA1, and GC [11].

DEPs associated with the regulation of coagulation included SERPINC1, FGB, FGG, HRG, ANXA1, and MYH9. SERPINC1 inhibits leukocyte and platelet recruitment to the post-ischemic retina, subsequently exerting a neuroprotective effect against retina ischemia–reperfusion injury [12]. FGB and FGG are fibrinogen chains that could increase with increased retinal vascular permeability due to ischemia or inflammation [13,14]. HRG is a glycoprotein secreted from activated platelets. It ameliorates a septic condition by suppressing excess reactive oxygen species production from neutrophils, reducing tissue damage at inflammatory sites [15]. ANXA1 inhibits neutrophil activation, migration, and infiltration, limiting inflammation in diabetic complications [16]. A reduction in ANXA1 can lead to retinal ganglion cell apoptosis in the ischemia–reperfusion mouse model [17]. On the other hand, ANXA1 also showed pro-inflammatory effects. ANXA1 knock-out mice exhibited a reduced infiltration of T cells in the spinal cord in an autoimmune encephalomyelitis model [18]. MYH9 enhanced TLR4 expression, interacting with calpain in human platelets, which led to TLR4-induced platelet aggregation and organ injury in systemic inflammation [19,20]. Oxidative stress and inflammatory reaction had been identified as the pathogenesis of myopia in previous studies [21]. The increased expression of these inflammatory or oxidative-stress-related proteins in this study can also serve as the basis to support the hypothesis.

In the central retina, hub proteins such as PSMC6, PSMA1, PSMA8, PSMC2, and PSMD12 are known subunits of the proteasome endopeptidase complex [22], which is consistent with GO analysis of whole DEPs in the central retina (Figure S2). These were identified as DOWN-DEPs in the central retina (Table S1). The human proteasome gene family included these genes. The protein expressed by these genes constitute the 26S proteasome (www.genome.jp/kegg/, 6 December 2022). Proteasome degrades misfolded proteins in response to environmental stress [23]. Under oxidative stress, a switch in the predominant proteasome complex happens from 26S to 20S, leading to dissociation of the 26S proteasome [24]. Based on these previous studies, we supposed that these changes in the central retina could be associated with oxidative stress.

Notably, the far-periphery retina showed the most significant change after myopia induction despite form deprivation rather than lens-induced myopia induction. Lens-induced myopia induction develops peripheral hyperopic defocus; however, form deprivation methods provide blur imaging to the whole retina. Both involve a reduction in spatial contrast, whereas lens-induced myopia may also involve a pathway initiated by the sign of defocus signal [25]. Form deprivation reduced the spatial contrast, leading to eye growth [26]. Therefore, we concluded that a far-periphery retina played a crucial role in response to the reduction in spatial contrast compared with the other retinal regions. Juan et al. reported that infant rhesus monkeys with form deprivation myopia exhibited relative peripheral hyperopia [27]. In human studies of young adults, relative peripheral hyperopia preceded adult-onset myopia [28]. This study cannot elucidate how the change in peripheral retina affects the central axial elongation. However, assuming that both central and far-periphery retina were under oxidative stress based upon our study, we concluded that the retinal response to oxidative stress could be region-specific in the myopia progression.

There are a few limitations to this study. First, this study was performed in a rabbit retina. Therefore, the result of this study cannot directly be applied to the pathogenesis of myopia in humans. However, the central fovea in humans has a high concentration of macular pigments, such as lutein and zeaxanthin, acting as powerful antioxidants and anti-inflammatory agents [29]. This protective mechanism against oxidative stress in the central retina is similar to that of the visual streak in the rabbit retina which showed lower oxygen consumption [30,31]. Second, the effect of retinal change on scleral remodeling cannot be elucidated based on the result of this study, which brings consequent axial elongation in myopia. The signaling pathway between the retina and scleral should be investigated further. Third, we did not measure the difference in oxidative stress for each retinal region. This should be measured with proteomics analysis, and the correlation between oxidative stress and regional protein expression change will be analyzed in a future study. Fourth, we did not validate the expression of hub proteins. We focused on revealing the difference of mechanism between each retinal region. In addition, because the retinal tissue was divided into three regions, the amount of retinal tissue was not sufficient. Validation of hub proteins and their related pathway will be performed in a following study.

In conclusion, the results of this study reinforced the importance of the far-periphery retina as the key responder to the reduction in spatial contrast caused by form deprivation-induced myopia based on bioinformatics. In addition, it is suggested that oxidative stress on the retina could be associated with the pathogenesis of myopia. Antithrombin-III (SERPINC1), fibrinogen gamma chain (FGG), and fibrinogen beta chain (FGB) are potential novel biomarkers for myopia. Based on this study, continuous changes in choroid and sclera in myopia should be more region-specifically studied in an animal model and humans, leading to identifying therapeutic clues for the progression of myopia.

## 4. Materials and Methods

### 4.1. Animals

We obtained 5 6-week-old New Zealand white rabbits (mean body weight, 1021–1240 g) from the Department of Laboratory Animal Resources, Yonsei Biomedical Research Institute, Yonsei University College of Medicine. All the animals were raised under a controlled temperature of 25 °C on 12 h light and dark cycle, with lights on at 9:00. Food and water were freely available. The experimental protocol was approved by the Animal Care and Ethics Committee of Yonsei Biomedical Research institute.

### 4.2. Myopia Induction Using Form Deprivation Method

The right eyes of all the rabbits were assigned as the myopia group, with the left eyes as the control group. Myopia was induced using the form deprivation method with tarsorrhaphy. Under general anesthesia with zolpidem (0.3 mL/kg, Virbac South Korea, Seoul, Republic of Korea) and rompun (0.2 mL/kg, Bayer Animal Health, Hanover, NJ, USA), local anesthesia was administered on the upper and lower eyelid margin using xylocaine. Upper and lower eyelids were split along the gray line. The epithelial layer on the gray line was removed with bipolar cauterization. The inner layer was approached and sutured with 6-0 vicryl (Ethicon, Raritan, NJ, USA). The outer layer was approached and sutured with 5-0 nylon (Ethicon, Raritan, NJ, USA). Eyelids were closed entirely, and the eyeball surface could not be observed grossly. Bleeding was controlled meticulously. Ofloxacin ointment (Santen, Nara, Japan) was applied. To prevent secondary infection and inflammation on the operation site, subcutaneous amoxicillin (7 mg/kg, Kyung Nam Pharm., Asan, Republic of Korea) and ibuprofen (2 mg/kg, Humedix, Seongnam, South Korea) were injected immediately after the operation. The wound site was dressed with 0.9% normal saline, and ofloxacin ointment was applied once a day for 7 days. Subcutaneous amoxicillin was injected once a day for 7 days. After 7 days, the nylon suture on the outer layer was removed. The closed eyelids were thus maintained for 6 weeks. When the tarsorrhaphy site was ruptured, and the eyeball surface was exposed, re-suture was performed immediately. After 6 weeks, the closed eyelid was split under general anesthesia.

### 4.3. Measurements of Ocular Biometrics

All measurements were performed under general anesthesia induced with zolpidem (0.3 mL/kg) and rompun (0.2 mL/kg). Measurements of the ocular biometry were taken with A-scan ultrasonography (UD-6000, TOMEY, Nagoya, Japan). After topical anesthetic use of proparacaine hydrochloride 0.5% (Hanmi, Seoul, Republic of Korea), applanation of the probe on the cornea was performed. Proper alignment of the probe was defined as strong ultrasonic wave reflection peaks of lens surfaces and retinas, which stood high from the level cursor line position. More than seven consecutive measurements of ocular components, including axial length, depth of the anterior segment, and lens thickness, were recorded and averaged after proper alignment was obtained. If the standard deviation of measurements was more than 0.04 mm, we repeated the measurements.

Cycloplegic refractions were performed using a streak retinoscope operated by an experienced ophthalmologist, who was unaware of which eye was induced and the purpose of the study. Three measurements with two axes of each were averaged as the spherical equivalent of all eyes, with readings reported to the nearest 0.25 D. Measurements were performed before the induction of myopia and after 6 weeks of myopia induction just before sacrifice.

### 4.4. Preparation of Retinal Tissue Sample

**Figure 6:**
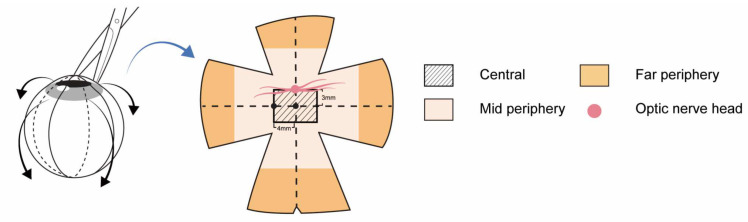
Preparation of retinal tissue sample after myopia induction. The retina was divided into three regions: central, mid-periphery, and far-periphery retina. Before the excision of the cornea, marking was performed along the limbus at 1, 4, 7, and 11 o’clock. Then, the eyeball wall was excised along the line from the marking spot to the optic nerve into a petaloid shape. The central retina was defined as the rectangular area bordered by the point at a distance of 4 mm in width and 3 mm in length on both sides from the point, which was ventral 3 mm from the optic nerve head. After separating the central retina, the remaining retina was divided evenly into two parts. The inner portion was defined as the mid-periphery retina, and the outer portion was defined as the far-periphery retina.

After measurements of ocular biometrics at 6 weeks of myopia induction, a lethal dose of KCL (JW Pharm., Seoul, Republic of Korea) was administered. The eyes were enucleated immediately after death, and the globe was separated from residual orbital tissue (Figure 6). Marking was performed at 1, 4, 7, and 11 o’clock along the limbus. The cornea was excised along the limbus. Additionally, the lens was extracted, and the vitreous was dissected. The eyeball was split along from the marked spot to the optic nerve. The eyeball wall was split into a petaloid shape (Figure 1A). The rabbit’s retinas had visual streaks corresponding to the human macula and had the highest density of cone and rod photoreceptors [32]. It was known to be located at about 3 mm ventral to the optic nerve head [33]. Therefore, we defined “central retina” as the rectangular area bordered on the point at a distance of 4 mm in width and 3 mm in length on both sides from the point, which was ventral 3 mm from the optic nerve head (Figure 6). After separating the central retina, each retina of the four petaloid parts was divided evenly into two parts. The inner portion was defined as the mid-periphery retina, and the other was defined as the far-periphery retina (Figure 6). The remaining vitreous was removed meticulously with fine forceps from the retinal surface. Retinal tissue was dissected from the choroid at each part of the retina. Six groups of retinal tissues were prepared: (1) control central, (2) control mid-periphery, (3) control far-periphery, (4) myopia central, (5) myopia mid-periphery, and (6) myopia far-periphery (Figure 6). The retinal tissue was stored at −70 °C until the mass spectrophotometric assay was performed.

### 4.5. In-Solution Digestion 

Proteins from pooled retinal tissues (100 μg) were digested into peptides by in-solution digestion. Briefly, 8 M urea in 100 mM ammonium bicarbonate (Sigma, St. Louis, MO, USA) was added to each pooled sample, and the mixture was homogenized using a disposable homogenizer (BiomesherII, Optima, Japan) until the tissues were no longer lumped. The samples were then briefly vortexed and sonicated on ice for 10 s. Samples were incubated for 20 min at room temperature and then centrifuged at 14,000 rpm for 15 min. Protein concentration of the supernatant was determined in triplicate using the micro BCA protein assay according to the manufacturer’s protocol (ThermoScientific, Rockford, IL, USA); then, 10 mM dithiothreitol (DTT, Sigma) for reduction and 30 mM iodoacetamide (IAA, Sigma) for alkylation were used to denature the proteins. Trypsin (ThermoScientific, Rockford, IL, USA), was added to the samples (1:50 = trypsin:sample) and incubated at 37 °C overnight. The activated trypsin reaction was quenched with 0.4% TFA (trifluoroacetic acid, ThermoScientific, Rockford, IL, USA), and peptides were desalted with a C_18_ Harvard macro spin column. The resulting peptides were dried and stored at −80 °C.

### 4.6. Protein Identification Using LC-MS/MS

Peptides were re-suspended in 0.1% formic acid (FA) in water and analyzed using the Q Exactive Orbitrap Hybrid Mass Spectrometer coupled with the EASY-nLC 1000 system (ThermoScientific, Bremen, Germany). For global proteome profiling analysis, the gradient was as follows: from 5% to 50% of solvent B for 85 min, from 50% to 80% of solvent B for 1 min, holding at 80% of solvent B for 8 min, and equilibrating the column at 1% of B for 30 min (Sol A: 0.1% FA in water, Sol B: 0.1% FA in acetonitrile). The peptides were eluted through a trap column and ionized through an EASY-spray column (50 cm × 75 μm ID) packed with 2 μm C_18_ particles at an electric potential of 1.8 kV. Complete MS data were acquired in a scan range of 400–2000 Th at a resolution of 70,000 at *m*/*z* 200, with an automated gain control target value of 1.0 × 10^6^ and a maximum ion injection of 100 ms. The maximal ion injection time for MS/MS was set to 100 ms at a resolution of 17,500. The dynamic exclusion time was set to exclude previously sequenced peaks for 30 s within a 10 ppm isolation window, and higher-energy collisional dissociation fragmentation was conducted under 30% of normalized collision energy. The proteomics data have been deposited to the ProteomeXchange consortium (http://proteomecentral.proteomexchange.org, accessed on 6 December 2022) with dataset identifier PXD038552 via the PRIDE partner repository [34].

### 4.7. Raw Data Processing and Statistical Analysis for Proteomics Data

Raw files were searched using the MaxLFQ algorithm integrated with MaxQuant software (v. 1.6.17.0, Max-Planck-Institute of Biochemistry, Planegg, Germany) [35] against the Uniprot rabbit proteome database (downloaded on June 2021). Protein identification was performed using at least one unique peptide, and only unique peptides, including those modified by the carbamidomethylation of cysteine, N-acetylation, and the oxidation of methionine. A false discovery rate cutoff of 1% was applied at the peptide spectrum match and protein levels. All LFQ intensities were log2-transformed to reduce the effect of outliers. To remove biological and technical biases, sum normalization was performed on the data using the relative protein abundance. Proteins that displayed a missing value in at least one group were filtered out to improve the confidence level.

Statistical and bioinformatics analyses for proteomics data were conducted based on the normalized protein abundance using MetaboAnalyst software version 5.0, a web-based tool for quantitative data analysis (downloaded on 1 June 2021). Clustering analysis, including PCA and hierarchical clustering using “Euclidean distances” and “ward” linkages, was presented to show the variance and differences among samples. Proteins with an expression greater than ±2-fold change and a *p*-value of less than 0.05 from the Student’s *t*-test in LFQ intensity were classified as DEPs.

### 4.8. Enrichment Analysis Using GO and Network Analysis

The gene ontology biological process and cellular component terms associated with total identified protein and DEPs were performed using the Database for Annotation, Visualization and Integrated Discovery and g:Profiler online tool [36]. Functional annotation clustering and KEGG pathway mapping were also performed. Protein networks and interactomes of DEPs were interrogated from the STRING 9.1 public database and visualized in Cytoscape. ClueGO (ver. 2.5.9, https://apps.cytoscape.org/apps/cluego, accessed on 1 October 2022), a Cytoscape plugin, was used to analyze and visualize the functionally organized GO terms. The analysis was based on Uniprot GO annotation. 

### 4.9. Screening of Hub Proteins

CytoHubba (ver. 0.1, https://apps.cytoscape.org/apps/cytohubba, accessed on 1 October 2022), a plugin for Cytoscape, provides 11 topological algorithms for detecting hub genes. Among all the algorithms, the MCC score performs better in hub gene prediction [37]. The MCC score of each node selected in network analysis was calculated, and the top 10 MCC values were classified as hub proteins. To analyze the GO of hub proteins, nodes with an MCC score of 10 or more were included in the GO analysis.

### 4.10. Statistical Analysis

Statistical analyses of the clinical data were performed using SPSS version 21.0 (IBM Corp., Armonk, NY, USA). The Kolmogorov–Smirnov test was used to confirm the normality of the data. To statistically compare data between groups, we used the Mann–Whitney U test or Wilcoxon signed-rank test for non-normally distributed data. We used the repeated measures ANOVA test to analyze repeated measured data between groups. With the assumption of sphericity with repeated measures ANOVA, Greenhouse–Geisser correction was used. A *p*-value of less than 0.05 was considered statistically significant in all the statistical tests.

## Data Availability

Data are available via ProteomeXchange with the identifier PXD038552.

## Supplementary Materials

The following supporting information can be downloaded at: https://www.mdpi.com/article/10.3390/ijms24021286/s1, Figure S1: The expression pattern of DEPs in each retinal region; Figure S2: Gene Ontology analysis of upregulated and downregulated differentially expressed proteins in each retinal region; Table S1: List of differentially expressed proteins in each retinal region after myopia induction.
