# Supplementary material for: Retinal Proteome Analysis Reveals a Region-Specific Change in the Rabbit Myopia Model
Source: Int J Mol Sci. 2023 Jan 9;24(2):1286. doi: 10.3390/ijms24021286 (PMC9863771; doi:10.3390/ijms24021286)
Supplement: Supplementary file 1 [file ijms-24-01286-s001.zip › Table S1 List of differentially expressed proteins in each retinal region after myopia induction.pdf]

Supplementary Table S1. List of differentially expressed proteins in each retinal region after myopia induction.

A. Central

Accession No.	Gene Name	Protein Name	Fold Change
**G1SKM2**	FBN1	Fibrillin 1	164.03
**U3KMR2**	AHSG	Alpha-2-HS-glycoprotein	16.34
**G1U8M6**	ATP1A2	Sodium/potassium-transporting ATPase subunit alpha	11.00
**G1U754**	HRG	Histidine-rich glycoprotein	8.93
**A0A5F9CM74**	CRYL1	Lambda-crystallin	8.40
**G1TVS4**	TRIM3	Hemopexin	6.05
**A0A1Y1B8B8**		IgG ligh chain	5.99
**A0A5F9C4X5**	NECTIN1	Nectin cell adhesion molecule 1	5.73
**B7NZP9**	FLNA	Filamin A, alpha isoform 2 (Predicted)	5.67
**A0A5F9CWI9**	GNB4	G protein subunit beta 4	4.11
**G1STF7**		Beta-1 metal-binding globulin	2.98
**G1U9S2**	ALB	Albumin	2.83
**A0A5F9DVE1**	TPM4	Tropomyosin 4	2.82
**G8ZF10**	HBA1	Alpha-globin 1	2.37
**G1TE96**	LANCL1	LanC like 1	2.34
**G1TPZ1**	LGALS1	Galectin	2.19
**G1TD51**		Histone H4	0.49
**G1TUE1**	ATP1B1	Sodium/potassium-transporting ATPase subunit beta	0.49
**A0A5F9DTD6**	C1orf43	Chromosome 1 open reading frame 43	0.48
**G1TJR7**		Histone H3	0.48
**A0A5F9DCB4**	SH3GL2	SH3 domain containing GRB2 like 2, endophilin A1	0.47
**G1TMV1**		Actin-depolymerizing factor	0.47
**G1U522**	PRKAR2A	Protein kinase cAMP-dependent type II regulatory subunit alpha	0.47
**G1SZ00**	CSRP1	Cysteine and glycine rich protein 1	0.46
**G1SVV6**	MAP2K1	Dual-specificity mitogen-activated protein kinase kinase 1	0.46
**G1SDA2**	CRABP1	Cellular retinoic acid-binding protein 1	0.46
**G1SIB9**	PYGB	Alpha-1,4 glucan phosphorylase	0.46
**G1TKV4**		Histone H3	0.46

**G1SPF5**		Uncharacterized protein	0.44
**G1T301**	ZNF326	Zinc finger protein 326	0.44
**G1U2G6**	PCBP4	Poly(rC) binding protein 4	0.44
**G1T892**	MAPRE3	Microtubule associated protein RP/EB family member 3	0.44
**G1SWI7**	PSMA8	Proteasome 20S subunit alpha 8	0.43
**G1T0H8**	PFKP	ATP-dependent 6-phosphofructokinase	0.43
**G1U6B2**	ALAD	Delta-aminolevulinic acid dehydratase	0.42
**G1SP71**	GNAT2	G protein subunit alpha transducin 2	0.42
**B6RFK9**	ATP2A2	Calcium-transporting ATPase	0.42
**G1TXQ6**		Uncharacterized protein	0.42
**G1T2I5**	RTN4	Reticulon	0.42
**A0A5F9C920**	PCP4	Purkinje cell protein 4	0.42
**G1SDF2**	PCMT1	Protein-L-isoaspartate O-methyltransferase	0.41
**G1TEH2**	ATP6V1C1	V-type proton ATPase subunit C	0.40
**A0A5F9CP41**		Ubiquitin-like domain-containing protein	0.40
**G1TU13**		40S ribosomal protein S17	0.38
**G1SNQ9**	NCEH1	Neutral cholesterol ester hydrolase 1	0.38
**A0A5F9CC60**	DNM3	Dynamin GTPase	0.37
**U3KMC7**	IMPG2	Interphotoreceptor matrix proteoglycan 2	0.36
**G1SYV0**	PSMC2	26S proteasome AAA-ATPase subunit RPT1	0.36
**G1TE69**		Serine/arginine-rich splicing factor 1	0.35
**G1T4C2**	DNM1	Dynamin GTPase	0.35
**G1U5M7**		Ribonucleoprotein	0.35
**G1T6D4**	PSMD12	Proteasome 26S subunit, non-ATPase 12	0.34
**G1U2E6**	PHB2	Prohibitin	0.33
**A0A5F9D2K9**	PFKM	ATP-dependent 6-phosphofructokinase	0.33
**G1SHS7**	WDR1	WD repeat domain 1	0.32
**G1T824**		Thymosin beta	0.31
**A0A5F9DQ17**	CMAS	CMP-N-acetylneuraminic acid synthase	0.30
**A0A5F9CJ60**	BCAT1	Branched-chain-amino-acid aminotransferase	0.30
**T2HVY9**	AKR1C32	3alpha/17beta/20alpha-hydroxysteroid dehydrogenase	0.29

**G1T9U6**	CALB2	Calretinin	0.29
**G1T336**	DDX3X	RNA helicase	0.29
**A0A5F9CEX6**	GSTZ1	Maleylacetoacetate isomerase	0.28
**G1U724**	SAE1	SUMO1 activating enzyme subunit 1	0.28
**G1SDG8**		Nucleolar and coiled-body phosphoprotein 1	0.25
**G1TCC1**	SLC16A1	Monocarboxylate transporter 1	0.23
**G1SFI2**		Protein S100	0.22
**G1T3S1**	PSMC6	Proteasome 26S subunit, ATPase 6	0.22
**G1TF14**	TMSB10	Thymosin beta	0.22
**A0A5F9C0X9**	API5	Apoptosis inhibitor 5	0.21
**A0A5F9D7H9**		Dynactin subunit 1	0.21
**G1TDI0**		Peptidase_M24 domain-containing protein	0.21
**G1SNT1**	EIF3J	Eukaryotic translation initiation factor 3 subunit J	0.21
**U3KM71**		ATP synthase subunit	0.19
**A0A5F9C5X9**	EIF4B	Eukaryotic translation initiation factor 4B	0.19
**A0A5F9CFK1**		Small nuclear ribonucleoprotein-associated protein	0.19
**A0A5F9D0X4**	NAPA	NSF attachment protein alpha	0.18
**G1SDA8**	PSMA1	Proteasome subunit alpha type	0.18
**G1T694**	PDE6A	Phosphodiesterase	0.17
**G1SXQ0**	GSTM3	Glutathione S-transferase	0.16
**G1TTH8**	IGSF8	Immunoglobulin superfamily member 8	0.16
**G1STG8**	GPM6A	Glycoprotein M6A	0.16
**A0A5F9CRJ4**		Dynamin-type G domain-containing protein	0.08
**A0A5F9C492**	CAMK2G	Calcium/calmodulin-dependent protein kinase	0.07

B. Mid-periphery

Accession No.	Gene Name	Protein Name	Fold Change
**G1TCC1**	SLC16A1	Monocarboxylate transporter 1	5.80
**A0A5F9CST3**	NUMA1	Nuclear mitotic apparatus protein 1	5.61
**G1T310**	PRXL2A	Peroxiredoxin-like 2 activated in M-CSF stimulated monocytes	4.36

**A0A5F9D776**	PTBP2	Polypyrimidine tract binding protein 2	3.83
**A0A5F9DBU9**		Elongation factor 1-gamma	3.78
**A0A5F9DME8**		Uncharacterized protein	3.14
**B7NZB4**	CDH2	Cadherin-2 (Fragment)	3.13
**A0A5F9DR44**		Uncharacterized protein	3.12
**A0A5F9D9E8**	GPHN	Molybdopterin molybdenumtransferase	3.09
**A0A5F9C9X1**	COPS4	COP9 signalosome complex subunit 4	3.05
**A0A5S8I1N0**	PPP2R2A	Serine/threonine-protein phosphatase 2A 55 kDa regulatory subunit B	2.94
**G1TCC8**	BCAS2	Pre-mRNA-splicing factor SPF27	2.69
**G1T542**	SNCG	Gamma-synuclein	2.65
**A0A5F9DH17**		Septin-type G domain-containing protein	2.44
**G1SI86**	S100A1	Protein S100	2.42
**G1TLQ8**	PSMC3	Proteasome 26S subunit, ATPase 3	2.41
**G1TH33**	ETFB	Electron transfer flavoprotein subunit beta	2.23
**G1T0T5**	COPS7A	COP9 signalosome subunit 7A	2.20
**A0A5F9C6U3**	RAB3D	RAB3D, member RAS oncogene family	2.13
**A0A5F9C870**		Uncharacterized protein	2.12
**A0A5F9CCA2**	RPN2	Dolichyl-diphosphooligosaccharide--protein glycosyltransferase subunit 2	2.01
**G1SM91**	FAH	Fumarylacetoacetase	0.48
**A0A5F9C1F9**	IMPG1	Interphotoreceptor matrix proteoglycan 1	0.48
**A0A1Y1B8B8**		IgG light chain	0.44
**G1SDD0**	ECHS1	Enoyl-CoA hydratase, short chain 1	0.41
**G1TM60**	NDUFA9	NADH:ubiquinone oxidoreductase subunit A9	0.40
**A0A5F9C8L5**	DDX5	DEAD box protein 5	0.40
**G1SQW0**	MTCH2	Mitochondrial carrier 2	0.39
**A0A5F9CDJ4**	LYPLA1	Acyl-protein thioesterase 1	0.37
**A0A5F9CPV4**	ALG8	ALG8 alpha-1,3-glucosyltransferase	0.36
**A0A5F9CEW8**	FMC1-LUC7L2	FMC1-LUC7L2 readthrough	0.34
**G1TD91**	NUDT21	Cleavage and polyadenylation specificity factor subunit 5	0.33
**Q8HY50**	TH	Tyrosine hydroxylase	0.32
**G1SYN3**	LZTFL1	Leucine zipper transcription factor-like protein 1	0.30

**A0A5F9D3B6**	ME2	Malic enzyme	0.29
**G1U6B2**	ALAD	Delta-aminolevulinic acid dehydratase	0.28
**Q5QSJ2**	cryab	HspB5 protein (Fragment)	0.23
**A0A5F9CM74**	CRYL1	Lambda-crystallin	0.13
**G1TQG1**	NDUFB9	Complex I-B22	0.08

C. Far-periphery

Accession No.	Gene Name	Protein Name	Fold Change
**B7NZH1**	DES	Desmin (Predicted)	34.50
**Q2PPJ8**	apoA-I	Apolipoprotein A-I	30.61
**G1T2H9**	COCH	Cochlin	24.82
**G1TT06**	SELENBP1	Selenium binding protein 1	23.97
**A0A5F9DHV4**	FGG	Fibrinogen gamma chain	23.24
**U3KML1**	BGN	Biglycan	23.10
**A0A5F9D3P7**	FGB	Fibrinogen beta chain	16.41
**G1TIS5**	ANXA1	Annexin	16.04
**A0A5F9DNV0**		C4a anaphylatoxin	14.65
**Q8HZ48**	Thbd	Thrombomodulin	14.44
**Q5QSJ2**	cryab	HspB5 protein (Fragment)	14.34
**A0A5F9DVP3**	COL14A1	Collagen type XIV alpha 1 chain	13.22
**U3KMR2**	AHSG	Alpha-2-HS-glycoprotein	12.38
**G1SP97**	LUM	Lumican	11.55
**G1SIJ2**	ACAT1	Acetyl-CoA acetyltransferase 1	10.49
**G1THZ6**	IGHM	Immunoglobulin heavy constant mu	9.92
**A0A1Y1B8B8**		IgG light chain	9.54
**A0A5F9DQW4**	IGHM	Immunoglobulin heavy constant mu	8.98
**G1SI83**	S100A13	S100 calcium binding protein A13	8.85
**G1TZB5**	ZNF34	60S ribosomal protein L8	8.57
**G1U754**	HRG	Histidine-rich glycoprotein	8.56
**G1SDD0**	ECHS1	Enoyl-CoA hydratase, short chain 1	8.55

**G1TVS4**	TRIM3	Hemopexin	8.52
**G1SU82**	GC	Gc-globulin	7.43
**G1TQG1**	NDUFB9	Complex I-B22	7.27
**G1TV79**		Collagen-binding protein	7.07
**A0A5F9CAW5**	Tpm2	Beta-tropomyosin isoform 1	6.66
**G8ZF10**	HBA1	Alpha-globin 1	6.58
**G1SXR1**	PRELP	Proline and arginine rich end leucine rich repeat protein	6.23
**A0A5F9DRS0**	LMNA	Lamin A/C	5.93
**G1TFV7**	SERPINA1	SERPIN domain-containing protein	5.32
**A0A5F9CDL7**	CAPG	Macrophage-capping protein	5.12
**G1T3V2**	HSPB1	Heat shock 27 kDa protein	4.84
**G1TSY8**	KIF12	Alpha-1-microglobulin	4.81
**G1SEK8**	FETUB	Fetuin B	4.74
**A0A5F9DVE1**	TPM4	Tropomyosin 4	4.63
**B8K174**	HBB	Hemoglobin, beta (Predicted)	4.51
**G1TES6**	HSD17B10	Hydroxysteroid 17-beta dehydrogenase 10	4.47
**G1TA37**		Uncharacterized protein	4.42
**A0A5F9D4K6**	PDLIM5	PDZ and LIM domain 5	4.37
**A0A5F9CKN8**	CRYBB2	Beta-B2 crystallin	4.16
**G1STF7**		Beta-1 metal-binding globulin	4.10
**G1TPZ1**	LGALS1	Galectin	4.06
**G1SFV1**	PDIA4	Protein disulfide-isomerase	4.05
**G1U5B3**	PSAP	Prosaposin	4.04
**A0A5F9D6U8**		Tropomyosin 1	4.01
**G1TM55**		40S ribosomal protein S6	3.95
**A0A5F9CE17**		Protein S100-A6	3.92
**G1SQW0**	MTCH2	Mitochondrial carrier 2	3.86
**A0A5F9DB63**	TLN1	Talin 1	3.74
**G1TBU9**	ACAA2	Acetyl-CoA acyltransferase 2	3.60
**G1SIK0**	SERPINC1	Antithrombin-III	3.46
**A0A5F9DH18**		Peptidase S1 domain-containing protein	3.43

**G1U4G9**		Chloride intracellular channel protein	3.37
**A0A5F9D4S1**	ACTN4	Actinin alpha 4	3.30
**A0A5F9DDG6**	GSTM2	Glutathione transferase	3.28
**A0A5F9CYK3**	HP1BP3	Heterochromatin protein 1-binding protein 3	3.28
**Q07298**		Alpha-1-antiproteinase S-1	3.28
**G1SU45**		Aldedh domain-containing protein	3.15
**G1SXD6**		Complex I-B14.5a	3.12
**G1TKL2**	MYL6	Myosin light chain 6	3.02
**A0A5F9CMB7**	C9	Complement component C9	2.99
**G1TE68**	H2AC19	Histone H2A	2.91
**A0A5F9DB16**	PADI2	Protein-arginine deiminase	2.86
**A0A5F9CC66**	DCN	Decorin	2.81
**G1U9S2**	ALB	Albumin	2.78
**G1TEM5**		Uncharacterized protein	2.78
**A0A5S8HLQ6**	H1-4	Histone H1.4	2.76
**G1SW97**	IVD	Butyryl-CoA dehydrogenase	2.73
**G1TED6**	ANXA5	Annexin	2.67
**G1SE57**		Uncharacterized protein	2.66
**G1T8M9**	LRPPRC	Leucine rich pentatricopeptide repeat containing	2.66
**A0A5F9C573**	AKR7L	Aldo-keto reductase family 7 like (gene/pseudogene)	2.62
**A0A5F9DEV8**	ICE2	Annexin	2.61
**G1U7C6**	H1-3	H1.3 linker histone, cluster member	2.54
**G1U1S8**		Cholecystokinin-12	2.51
**G1SV04**	NDUFB11	Complex I-ESSS	2.46
**G1SL68**	MYH9	Myosin heavy chain 9	2.35
**A0A5F9D6M0**	KTN1	Kinectin 1	2.19
**G1T6D1**		60S ribosomal protein L23	2.18
**A0A5F9CZZ7**		RRM domain-containing protein	2.06
**A0A5F9D5J2**	OLA1	Obg like ATPase 1	2.02
**A0A5F9DFZ5**	VCL	Metavinculin	2.00
**G1SPN9**	ARL3	ADP ribosylation factor like GTPase 3	0.50

**G1TWX4**		CS domain-containing protein	0.50
**G1SRF7**	HSPA9	75 kDa glucose-regulated protein	0.50
**A0A5F9C3G4**	CAMK2D	Calcium/calmodulin-dependent protein kinase	0.49
**A0A5F9C8G6**	RALB	Small monomeric GTPase	0.49
**G1SCN8**	CCT3	T-complex protein 1 subunit gamma	0.48
**G1TJK2**	NEFL	Neurofilament light polypeptide	0.47
**G1SNQ9**	NCEH1	Neutral cholesterol ester hydrolase 1	0.47
**Q9GLM9**		Alpha-tubulin	0.47
**G1T0H8**	PFKP	ATP-dependent 6-phosphofructokinase	0.47
**G1SG42**	FAM98B	Family with sequence similarity 98 member B	0.47
**A0A5F9CFK1**		Small nuclear ribonucleoprotein-associated protein	0.47
**A0A5F9C4P5**	FABP7	Fatty acid binding protein 7	0.47
**A0A5F9DG35**	EPB41L3	Erythrocyte membrane protein band 4.1 like 3	0.47
**G1T7R2**		Tyrosine 3-monooxygenase/tryptophan 5-monooxygenase activation protein epsilon	0.46
**A0A5F9DVU2**	HK2	Hexokinase	0.45
**A0A5S8H2S7**	GOT2	Aspartate aminotransferase	0.45
**G1SGP1**	UQCRC1	Ubiquinol-cytochrome c reductase core protein 1	0.44
**G1U2G6**	PCBP4	Poly(rC) binding protein 4	0.44
**G1TTU6**		S-phase kinase-associated protein 1	0.44
**G1SEE0**	RBBP4	RB binding protein 4, chromatin remodeling factor	0.43
**A0A5F9DJ49**	ATOX1	Antioxidant 1 copper chaperone	0.43
**G1SNS5**		Rab GDP dissociation inhibitor	0.42
**G1SWI3**	VDAC2	Outer mitochondrial membrane protein porin 2	0.42
**U3KMC7**	IMPG2	Interphotoreceptor matrix proteoglycan 2	0.41
**A0A5F9D2K5**	PTER	Parathion hydrolase-related protein	0.41
**G1T140**	PACSIN1	Protein kinase C and casein kinase substrate in neurons protein 1	0.40
**G1TXG9**	NDUFS5	Complex I-15 kDa	0.39
**G1U522**	PRKAR2A	Protein kinase cAMP-dependent type II regulatory subunit alpha	0.39
**G1TMV1**		Actin-depolymerizing factor	0.38
**G1TM60**	NDUFA9	NADH:ubiquinone oxidoreductase subunit A9	0.38
**G1T567**	RHOA	Ras homolog family member A	0.37

**G1SZE0**	VPS35	Vacuolar protein sorting-associated protein 35	0.37
**G1TS42**	AGL	4-alpha-glucanotransferase	0.36
**A0A5F9CZI7**	HSPA4	Heat shock protein family A (Hsp70) member 4	0.36
**G1SIB9**	PYGB	Alpha-1,4 glucan phosphorylase	0.36
**G1TR82**	TUBA4A	Tubulin alpha chain	0.36
**G1T824**		Thymosin beta	0.36
**G1TKN4**	RAC1	Rac family small GTPase 1	0.33
**A0A5F9DCJ2**	MACROH2A1	MacroH2A.1 histone	0.33
**G1T2I5**	RTN4	Reticulon	0.32
**G1U5S4**		Tubulin beta chain	0.32
**G1SP71**	GNAT2	G protein subunit alpha transducin 2	0.32
**G1SQC0**	CNRIP1	CB1 cannabinoid receptor-interacting protein 1	0.32
**A0A5F9CFR1**	HNRNPDL	Heterogeneous nuclear ribonucleoprotein D like	0.32
**G1SDM3**	RDH12	Retinol dehydrogenase 12	0.32
**G1SDA8**	PSMA1	Proteasome subunit alpha type	0.30
**A7UJ11**	RS1	Retinoschisin	0.28
**G1SX37**	CFL2	Cofilin 2	0.28
**G1SHS7**	WDR1	WD repeat domain 1	0.28
**G1U4L4**		Uncharacterized protein	0.27
**G1TF26**	ROM1	Retinal outer segment membrane protein 1	0.26
**G1T013**	ME1	Malic enzyme	0.25
**A0A5F9D663**		Uncharacterized protein	0.25
**G1SVP6**	H2AC6	Histone H2A	0.24
**G1T845**	NSF	Vesicle-fusing ATPase	0.24
**A0A5F9C6Y6**	LAP3	Cysteinylglycine-S-conjugate dipeptidase	0.24
**A0A5S8H548**		Serine/threonine-protein phosphatase	0.22
**G1SN16**	APEX1	DNA-(apurinic or apyrimidinic site) endonuclease	0.21
**A0A5F9CRJ4**		Dynamin-type G domain-containing protein	0.21
**G1T2J6**	SLC25A12	Solute carrier family 25 member 12	0.21
**A0A5F9D3B6**	ME2	Malic enzyme	0.20
**G1SVA3**	PSMD11	Proteasome 26S subunit, non-ATPase 11	0.20

**A0A5F9C326**	GCLC	Glutamate--cysteine ligase	0.18
**G1TUE1**	ATP1B1	Sodium/potassium-transporting ATPase subunit beta	0.18
**A0A5F9CKT7**	TRA2A	Transformer 2 alpha homolog	0.18
**A0A5F9C4A7**	AP2B1	AP complex subunit beta	0.18
**A0A5F9DHZ7**	SLC3A2	4F2 cell-surface antigen heavy chain	0.17
**G1TXN1**	NIT2	Nitrilase family member 2	0.17
**G1SYC6**	HDGFL3	HDGF like 3	0.17
**G1TK32**		PFK domain-containing protein	0.16
**G1STB1**		Uncharacterized protein	0.15
**G1U5M7**		Ribonucloprotein	0.14
**G1T894**	BIN1	Bridging integrator 1	0.13
**U3KM71**		ATP synthase subunit	0.12
**G1SXQ0**	GSTM3	Glutathione S-transferase	0.11
**G1TDC3**		Gal_mutarotas_2 domain-containing protein	0.10
**G1T5Y1**	MAPK1	Mitogen-activated protein kinase	0.10
**A0A5F9CED6**	ATP6V1H	V-type proton ATPase subunit H	0.09
**G1SY68**	SF3B2	Splicing factor 3b subunit 2	0.08
**A0A5F9CZG3**		Eukaryotic translation initiation factor 5A	0.06
